# Headache and transient visual loss as the only presenting symptoms of vertebral artery dissection: a case report

**DOI:** 10.1186/s13256-016-0893-8

**Published:** 2016-04-25

**Authors:** Camille Yvon, Ashok Adams, Duncan McLauchlan, Conor Ramsden

**Affiliations:** 1 Institute of Ophthalmology, University College London, London, UK; 2 Cardiff University, Cardiff, UK; 3 Department of Radiology, Royal London Hospital, Barts Health NHS Trust, London, UK; 4 Moorfields Eye Hospital, London, UK

**Keywords:** Vertebral artery dissection, Stroke, Transient visual loss

## Abstract

**Background:**

Vertebral artery dissection is an important cause of stroke in the young and diagnosis is often challenging as symptoms are varied and subtle.

**Case presentation:**

A 33-year-old, previously healthy, white male office worker was stretching his neck when he developed sudden left-sided visual loss lasting 5 minutes associated with headache. He had no other neurological symptoms or signs. He was investigated with a computed tomography angiogram, which revealed a left vertebral artery dissection with a right posterior cerebral artery vascular occlusion.

**Conclusions:**

We describe an atypical case of vertebral artery dissection presenting with sudden transient visual disturbance without neurological signs in an otherwise healthy man. This is a rare but potentially fatal condition that can result in thromboembolic infarction. A high index of suspicion is crucial to make an early diagnosis and avoid devastating neurological outcomes.

## Background

Vertebral artery dissection (VAD) is an important cause of stroke in the young. Combined with cerebral arterial dissections (CADs), it may account for 25 to 30 % of ischemic strokes in patients below the age of 50 years [1, 2]. Diagnosis is often challenging for clinicians, as symptoms are varied and subtle. VAD is a potentially fatal condition that arises from a tear in the vessel wall leading to the formation of an intramural hematoma [1, 2]. Disruption of the vessel wall may result in thromboembolism and subsequent ischemic stroke. The vertebral arteries are prone to injury from neck extension, flexion, or rotation. VAD may occur following trivial neck trauma, such as prolonged stretching, chiropractic manipulation, or sporting injuries. Predisposing genetic factors and connective tissue diseases are also implicated in the pathophysiology, including Marfan syndrome, Ehlers–Danlos syndrome, and fibromuscular dysplasia [1, 2]. Here we report an unusual case of VAD without any clinical signs at the time of presentation.

## Case presentation

A 33-year-old, previously healthy, white male office worker was sitting at his desk and stretching his neck when he developed sudden left-sided visual loss lasting 5 minutes associated with headache. He had no past relevant medical or surgical history, and no previous use of medications or previous infectious symptoms. He did not smoke cigarettes. He presented to eye casualty and was noted to have a headache requiring analgesia but visual field testing by confrontation was normal. He had a Glasgow Coma Scale (GCS) of 15, a cranial nerve examination was otherwise normal, as were the power, tone, reflexes, coordination, and sensation in his peripheral nervous system. On review of his history he was unclear if his visual loss was monocular or binocular.

**Fig. 1:**
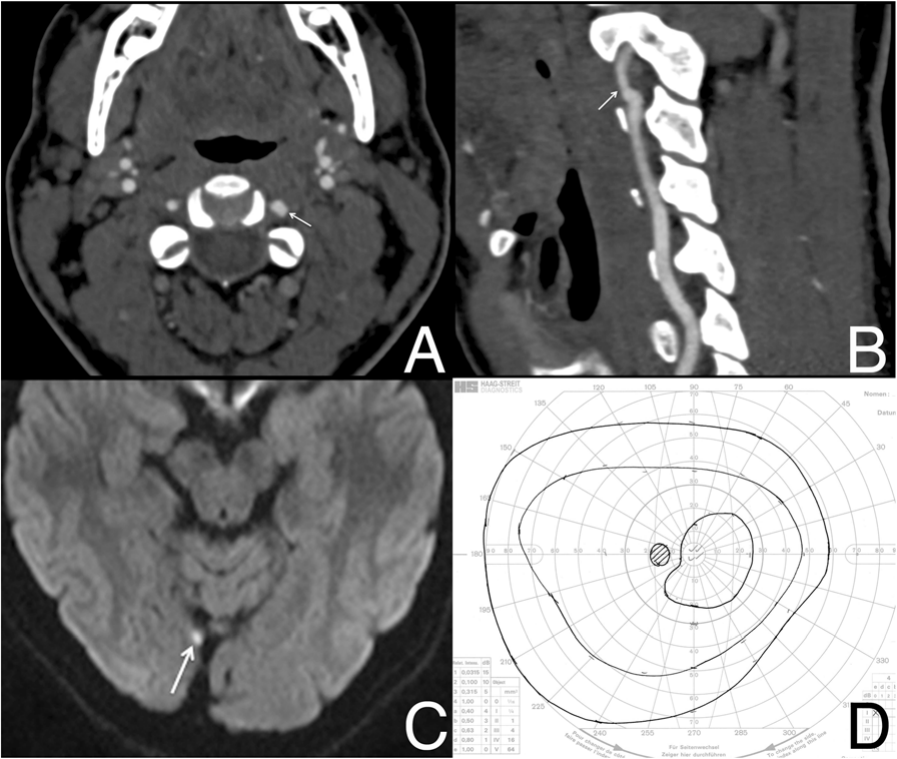
Diagnostic imaging and perimetry. **a** Computed tomography of head and neck (axial view) showing a mural irregularity in the V2 segment of the left (arrow) vertebral artery compared to the right. **b** Sagittal reformat showing the change in caliber of the lumen of the left vertebral artery (arrow). **c** Diffusion weighted (B1000) magnetic resonance image demonstrating the focal cortical infarct in the right occipital lobe (arrow). **d** Goldmann visual field showing no evidence of homonymous field defect

He was investigated with a computed tomography angiogram (CTA) covering his aortic arch to the circle of Willis in order to assess for arterial dissection. The initial report excluded a CAD and he was sent home. However, a subsequent review by a consultant neuroradiologist the following morning revealed a left VAD with a right posterior cerebral artery vascular occlusion (Fig. 1), the latter presumed to be an embolic sequel of the VAD. Our patient was then immediately contacted and admitted under the care of the stroke physicians and started on antiplatelet therapy (6 months of 75 mg clopidogrel). Six days later, he had a magnetic resonance imaging (MRI) and magnetic resonance angiography (MRA) of his head and neck, confirming the presence of a tiny focal cortical infarct within his right occipital lobe. Subsequent formal perimetry revealed no homonymous hemianopia.

## Discussion

The incidence of stroke in young adults is rising and it is increasingly important to consider stroke in younger patients with neurological symptoms [3]. We report an unusual case of VAD without any clinical signs at the time of presentation. Only after careful deliberation of the patient history was the decision made to perform neuroradiology and the diagnosis was subsequently made and treatment commenced, potentially avoiding further extension of the stroke and possible permanent sequelae.

Symptoms are variable and nonspecific, making diagnosis very difficult. They may include neck or head pain, partial Horner’s syndrome, and those of ischemic stroke in its involved territory [4]. Ophthalmic manifestations have previously been reported with variable frequency (15 to 86 %) [4, 5]. The most common visual symptoms comprise diplopia, blurred vision, or visual field defects [4, 5].

This case highlights the important role of radiologic imaging in diagnosis. Vertinsky *et al*. [6] show that CTA is superior to MRA in the detection of VAD. The small size of the vertebral arteries may make it difficult to distinguish initial stages of intramural hemorrhage (illustrated by a methemoglobin crescent sign) when using MRA.

Antithrombotic therapy is the preferred treatment option due to the underlying pathological mechanism of VAD. However, there is no robust randomized control data to confirm the therapeutic superiority of anticoagulants over antiplatelet agents [7]. In this case our patient was prescribed clopidogrel.

## Conclusions

We describe an atypical case of VAD presenting with sudden transient visual disturbance without neurological signs in an otherwise healthy man. This is a rare but potentially fatal condition that can result in thromboembolic infarction. It may be easily misdiagnosed or missed altogether because symptoms and signs are subtle, and the mechanism of injury is not always evident. A high index of suspicion is crucial to make an early diagnosis and avoid devastating neurological outcomes.

## Consent

Written informed consent was obtained from the patient for publication of this case report and accompanying images. A copy of the written consent is available for review by the Editor-in-Chief of this journal.

## Abbreviations

CAD: cerebral arterial dissection
CTA: computed tomography angiogram
GCS: Glasgow Coma Scale
MRA: magnetic resonance angiography
MRI: magnetic resonance imaging
VAD: vertebral artery dissection
